# Association between pre-pregnancy multimorbidity and adverse maternal outcomes: A systematic review

**DOI:** 10.1177/26335565221096584

**Published:** 2022-04-30

**Authors:** Hilary K Brown, Anthony McKnight, Amira Aker

**Affiliations:** 1 Department of Health & Society, 33530 University of Toronto Scarborough, Toronto, ON, Canada; 2 Dalla Lana School of Public Health, 7938 University of Toronto, Toronto, ON, Canada; 3 Women’s College Hospital, Toronto, ON, Canada

**Keywords:** Comorbidity, multimorbidity, pregnancy, severe maternal morbidity, maternal mortality, systematic review

## Abstract

**Objective:**

We reviewed the literature on the association between pre-pregnancy multimorbidity (co-occurrence of two or more chronic conditions) and adverse maternal outcomes in pregnancy and postpartum.

**Data sources:**

Medline, EMBASE, and CINAHL were searched from inception to September, 2021.

**Study selection:**

Observational studies were eligible if they reported on the association between ≥ 2 co-occurring chronic conditions diagnosed before conception and any adverse maternal outcome in pregnancy or within 365 days of childbirth, had a comparison group, were peer-reviewed, and were written in English.

**Data extraction and synthesis:**

Two reviewers used standardized instruments to extract data and rate study quality and the certainty of evidence. A narrative synthesis was performed.

**Results:**

Of 6,381 studies retrieved, seven met our criteria. There were two prospective cohort studies, two retrospective cohort studies, and 3 cross-sectional studies, conducted in the United States (n=6) and Canada (n=1), and ranging in size from n=3,110 to n=57,326,681. Studies showed a dose-response relation between the number of co-occurring chronic conditions and risk of adverse maternal outcomes, including severe maternal morbidity or mortality, hypertensive disorders of pregnancy, and acute health care use in the perinatal period. Study quality was rated as strong (n=1), moderate (n=4), or weak (n=2), and the certainty of evidence was very low to moderate.

**Conclusion:**

Given the increasing prevalence of chronic disease risk factors such as advanced maternal age and obesity, more research is needed to understand the impact of pre-pregnancy multimorbidity on maternal health so that appropriate preconception and perinatal supports can be developed.

## Introduction

The prevalence of chronic conditions such as diabetes mellitus, cardiovascular disease, asthma, and mental illness is steadily increasing worldwide, now accounting for 50% of the global disease burden.^1^ In industrialized countries, half of adults over 20 years have at least one chronic condition.^2^ The co-occurrence of two or more chronic conditions, i.e., multimorbidity, is a growing public health concern, with the prevalence increasing from 25% in 2003 to 32% in 2016.^3^ Multimorbidity was initially considered to be a phenomenon of aging.^4^ Yet, studies have shown that while multimorbidity risk increases with age, individuals are affected across the life course, with as many as 18% of 18 to 49-year-olds having multimorbidity.^3^ Growing evidence suggests individuals with multimorbidity account for a disproportionate amount of health care use and cost. In the general population, there is a dose-response relation between the number of chronic conditions and rates of hospitalization, risk of death, and magnitude of attributable health care costs.^5-7^ These patterns reveal gaps in primary care of persons with multimorbidity.

The impact of multimorbidity on maternal outcomes in pregnancy and postpartum is less understood. Increasing rates of maternal mortality,^8^ severe maternal morbidity,^8^ and acute perinatal health care use^9^ have prompted investigations into the role of chronic disease in explaining these outcomes. Studies from Canada,^10^ the United Kingdom,^11^ and the United States^12^ have consistently shown that chronic diseases, and in particular cardiovascular disease, chronic hypertension, and diabetes mellitus, are top contributors. Yet, while chronic conditions are prevalent and are known to co-occur, obstetric research and practice remain focused on the impact of single chronic conditions rather than on their combined impacts.^13^ Emerging evidence suggests this is an important oversight, with a number of studies from the United States, the United Kingdom, and Germany confirming that as many as one in five women enter pregnancy with two or more chronic conditions.^14-16^ High rates of adverse maternal outcomes among women with multimorbidity would indicate the need for better preventive efforts to address modifiable risk factors in the preconception period and patient-centered supports perinatally.

We performed a systematic review of the literature on the association between pre-pregnancy multimorbidity and adverse maternal outcomes in pregnancy and postpartum.

## Methods and materials

### Information sources and search strategy

This systematic review was prospectively registered in the PROSPERO database (ID: CRD42019137416). We used the Preferred Reporting Items for Systematic Reviews and Meta-Analyses (PRISMA)^17^ guidelines to inform our review. In consultation with a librarian, controlled vocabulary and plain language keywords were developed for the concepts of multimorbidity (e.g., multimorbidity, multiple chronic conditions)^18^ and adverse maternal outcomes (e.g., maternal mortality, severe maternal morbidity, perinatal hospital use) (Table 1). We searched Medline, EMBASE, and CINAHL from inception to September 14, 2021. One author performed the initial database search. Two authors independently reviewed the results and searched the bibliographies of selected studies to identify additional studies missed in the database search.

**Table 1.** Study quality.

Study	Study design	Selection bias	Confounding	Detection bias	Attrition bias	Overall rating
Admon 2018 a^27^	Weak	Strong	Weak	Moderate	Strong	Weak
Admon 2018 b^28^	Weak	Strong	Moderate	Moderate	Strong	Moderate
Bandoli 2017^29^	Moderate	Weak	Strong	Strong	Weak	Weak
Brown 2020^30^	Weak	Strong	Moderate	Moderate	Strong	Moderate
Czerwinski 2012^31^	Moderate	Moderate	Moderate	Strong	Weak	Moderate
Prophet 2018^32^	Moderate	Strong	Moderate	Moderate	Strong	Strong
Varner 2020^33^	Moderate	Strong	Weak	Moderate	Strong	Moderate

Notes: Quality related to study design is rated as moderate for cohort and case-control studies and weak for cross-sectional studies; Quality related to selection bias is rated as strong if the study sample is likely representative and the response rate is ≥ 80%, moderate if study sample is somewhat representative or response rate is 60-79%, and weak if the study sample is self-referred or volunteers or the response rate is < 60% or not reported; Quality related to control for confounding is rated as strong if ≥ 80% of confounders are controlled for, moderate if 60-79% of confounders are controlled for, and weak if < 60% of confounders are controlled for; Quality related to detection bias is rated as strong if clinical diagnoses are used, moderate if population-based data are used, and weak if self-reported data are used; Quality related to attrition bias is rated as strong if follow-up is ≥ 80% or the study is a retrospective cohort study or cross-sectional study, moderate if follow-up is 60-79%, and weak if follow-up is < 60% or not reported. Overall rating is weak if ≥ 2 components are rated as weak, moderate if 1 component is rated as weak, and high if 0 components are rated as weak.

### Eligibility criteria and study selection

Two authors independently reviewed titles and abstracts. To be included, studies were required to (1) examine, as an exposure, multimorbidity diagnosed before conception; (2) examine, as an outcome, any maternal medical complication, acute health care use, or death between conception and 365 days postpartum; (3) have a comparison group of women with no chronic conditions; (4) be peer-reviewed; and (5) be written in English. To be consistent with established multimorbidity frameworks, which define multimorbidity as the presence of ≥ 2 chronic conditions from among a list of several conditions,^18^ we excluded studies only examining “one or more” chronic conditions (i.e., not allowing differentiation of one vs. multiple conditions) and those in which the conditions examined were not chronic (e.g., those that included gestational diabetes or gestational hypertension). While we initially planned to exclude studies that examined a specific comorbidity (e.g., diabetes and depression) without further evaluation of other conditions, a preliminary review of our search results revealed very few studies examining multimorbidity from among a long list of possible chronic conditions; therefore, within the scope of the search strategy, where specific combinations of morbidities were identified, the outcomes for these studies were also reported. Last, we excluded studies that examined offspring outcomes only, since our focus was on maternal health.

### Data extraction

After establishing consensus on the list of eligible studies via full text review, two authors independently extracted available study data using a standardized form, created *a priori* to reflect the elements of the Strengthening the Reporting of Observational Studies in Epidemiology statement.^19^ The form included: study location, study period, study design, data sources, sample size, response rates, follow-up rates, missing data rates, inclusion criteria, multimorbidity definition, outcome definition(s), and confounders controlled for. We also extracted outcome event rates and measures of effect, where available. Where information was unclear or missing, we contacted study authors by email for clarification. Discrepancies in data extraction were discussed by the authors until consensus was reached.

### Quality assessment

Two authors independently conducted a quality assessment of each article using the Effective Public Health Practice Project Quality Assessment Tool.^20^ Study quality was rated based on study design, risk of selection bias (representativeness of the cohort, response rate), control for confounding, risk of detection bias (assessment of the outcome), and risk of attrition bias (loss to follow-up). For control for confounding, we identified variables *a priori* that are associated with chronic disease risk and with adverse maternal outcomes: maternal age, socioeconomic status, race/ethnicity, and health behaviours (e.g., smoking, BMI). As in previous research,^21,22^ we rated studies overall as being weak (≥ 2 individual categories rated as weak), moderate (1 category rated as weak), or strong in quality (0 categories rated as weak).

### Data synthesis

We planned to use random effects models to calculate pooled unadjusted and adjusted odds ratios for the association between multimorbidity and adverse maternal outcomes. However, exposure and outcome definitions were not homogeneous for a sufficient number of studies to conduct a meta-analysis. We therefore described the study results using a narrative synthesis following Synthesis Without Meta-analysis (SWiM) guidelines.^23^

### Grading of the evidence

We used the Grading of Recommendations, Assessment, Development and Evaluations (GRADE) approach,^24^ modified for narrative syntheses,^25^ to ascertain the certainty of evidence. By default, studies started at low-certainty evidence and were then downgraded or upgraded based on *a priori* criteria. Criteria for downgrading evidence were methodological limitations as identified by the Effective Public Health Practice Project Quality Assessment Tool, indirectness (i.e., dissimilarity of research evidence from clinical practice in terms of population, exposure, or outcomes), imprecision (i.e., 95% confidence intervals including the null value, or a small number of events of < 400), inconsistency (i.e., differences in the magnitude of effects across studies), and likelihood of publication bias (i.e., the presence of only small positive studies). Criteria for upgrading were a large effect size (i.e., OR or RR > 2.0), a dose-response relationship between exposure and outcome, and attenuation by plausible confounders.^26^

## Results

### Study selection

**Figure 1.**
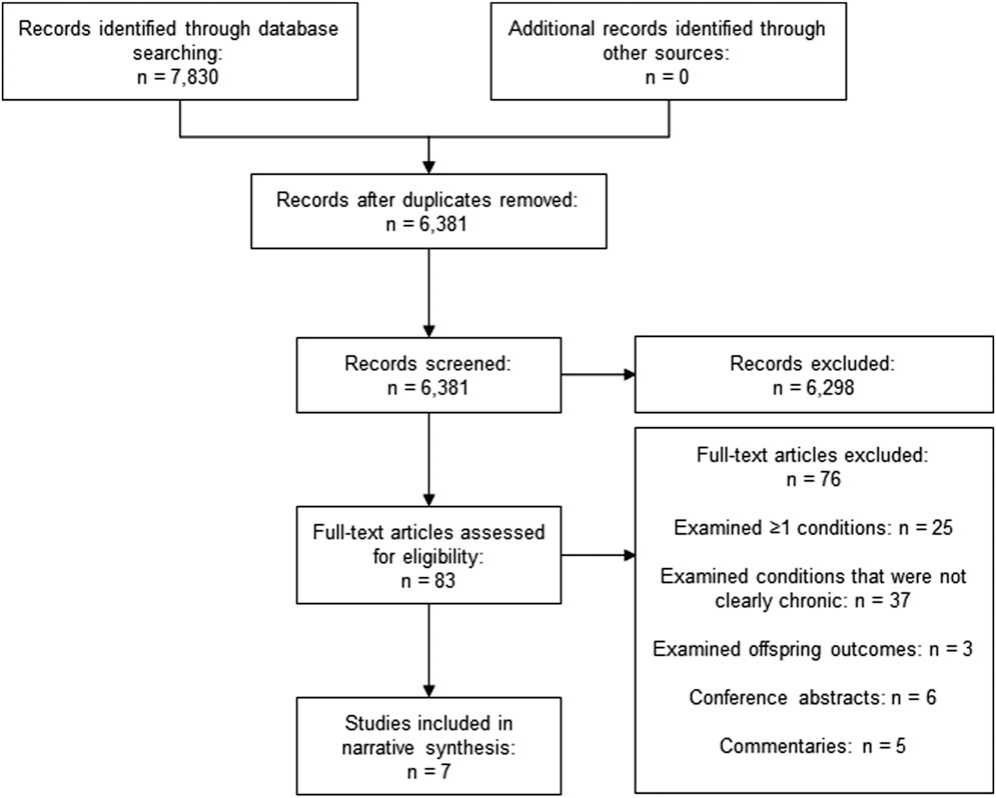
PRISMA flowchart.

Figure 1 shows the study selection process. Our database search revealed 6,381 unique studies, of which 6,298 were excluded due to lack of relevance. Eighty-three full-text articles were reviewed. Of these, we excluded studies that examined: (1) “one or more” chronic conditions (n=25), (2) conditions that were not clearly chronic (n=37), and (3) offspring outcomes only (n=3). We also excluded conference abstracts (n=6) and commentaries (n=5). After these exclusions, 7 studies met the inclusion criteria for our review.^27-33^

### Study characteristics

Study characteristics are described in Table 2. Six studies were conducted in the United States^27-32^ and one in Canada.^33^ There were two prospective cohort studies,^29,31^ two retrospective cohort studies,^32,33^ and three cross-sectional studies.^27,28,30^ Sample sizes ranged from 3,110^29^ to 57,326,681 women.^32^ Two studies defined multimorbidity as ≥ 2 chronic conditions from among a list of 8 to 16 eligible conditions;^27,28^ two studies used comorbidity indices applied to pre-pregnancy diagnoses (i.e., the Elixhauser Comorbidity Index and the Johns Hopkins Adjusted Clinical Group System Aggregated Diagnosis Groups [ADGs]),^30,33^ and three studies measured combinations of specific co-occurring conditions,^29,31,32^ better described as comorbidity (i.e., asthma and migraine; autoimmune disease and depression; sickle cell disease and HIV). Outcomes were severe maternal morbidity or maternal mortality (n=3);^27,28,30^ other composite measures of pregnancy complications (n=1);^32^ hypertensive disorders of pregnancy (n=2);^29,31,32^ and health services indicators of complications (n=2; i.e., perinatal emergency department visits, delivery hospitalization length of stay, and hospital transfer).^27,29^ All studies controlled for confounding to some extent.

**Table 2.** Study Findings.

Outcome	Study (details)	Exposure groups	% with outcome	OR or RR (95% CI)	aOR or aRR (95% CI)
Any pregnancy complication	Prophet 2018^27^	HIV + sickle cell disease	15.2	NR	1.89 (0.82-4.33)
		HIV only	8.4	NR	0.95 (0.89-1.01)
		Sickle cell disease only	9.8	NR	1.11 (1.03-1.18)
		Neither condition	8.1	[Referent]	[Referent]
Severe maternal morbidity or mortality	Admon 2018 a^22^	≥ 2 chronic conditions	6.4	NR	NR
		1 chronic condition	3.0	NR	NR
		No chronic conditions	1.7	[Referent]	[Referent]
	Admon 2018 b^23^ (non-Hispanic White)	≥ 2 chronic conditions	2.9	NR	NR
		1 physical condition	1.8	NR	NR
		1 behavioural condition	2.4	NR	NR
		No chronic conditions	1.3	[Referent]	[Referent]
	Admon 2018 b^23^ (non-Hispanic Black)	≥ 2 chronic conditions	5.5	NR	NR
		1 physical condition	3.2	NR	NR
		1 behavioural condition	4.0	NR	NR
		No chronic conditions	2.2	[Referent]	[Referent]
	Admon 2018 b^23^ (Hispanic)	≥ 2 chronic conditions	4.7	NR	NR
		1 physical condition	2.9	NR	NR
		1 behavioural condition	3.1	NR	NR
		No chronic conditions	1.6	[Referent]	[Referent]
	Admon 2018 b^23^ (Asian / Pacific Islander)	≥ 2 chronic conditions	5.4	NR	NR
		1 physical condition	2.8	NR	NR
		1 behavioural condition	3.6	NR	NR
		No chronic conditions	1.5	[Referent]	[Referent]
	Admon 2018 b^23^ (AI/AN)	≥ 2 chronic conditions	4.1	NR	NR
		1 physical condition	2.2	NR	NR
		1 behavioural condition	3.3	NR	NR
		No chronic conditions	2.0	[Referent]	[Referent]
	Brown 2020^25^	≥ 3 comorbidities	5.6	12.1 (11.5-12.7)	9.1 (8.7-9.6)
		2 comorbidities	3.8	8.1 (7.8-8.5)	6.6 (6.3-6.9)
		1 comorbidity	2.4	5.0 (4.8-5.2)	4.4 (4.2-4.6)
		0 comorbidities	0.5	[Referent]	[Referent]
	Brown 2020^25^ (no blood transfusion)	≥ 3 comorbidities	2.4	15.1 (14.0-16.3)	9.7 (8.9-10.5)
		2 comorbidities	1.1	6.6 (6.1-7.1)	4.8 (4.5-5.2)
		1 comorbidity	0.6	3.8 (3.6-4.1)	3.1 (2.9-3.3)
		0 comorbidities	0.2	[Referent]	[Referent]
	Preeclampsia and related conditions	Bandoli 2017^24^ (any preeclampsia)	Crohn’s disease + depression	NR	NR	NR
			Crohn’s disease	NR	NR	1.82 (1.01-3.30)
			Neither condition	NR	[Referent]	[Referent]
		Bandoli 2017^24^ (any preeclampsia)	Psoriasis + depression	NR	NR	1.39 (0.25-7.62)
			Psoriasis only	NR	NR	1.58 (0.86-2.89)
			Neither condition	NR	[Referent]	[Referent]
		Bandoli 2017^24^ (any preeclampsia)	Rheumatoid arthritis + depression	NR	NR	1.08 (0.21-5.55)
Rheumatoid arthritis only			NR	NR	1.65 (1.04-2.63)
Neither condition			NR	[Referent]	[Referent]
Czerwinski 2012^26^ (any preeclampsia)		Migraine + asthma	8.9	3.26 (1.52-6.99)	3.77 (1.61-8.82)
		Migraine only	3.8	1.32 (0.79-2.22)	0.89 (0.49-1.63)
		Asthma only	5.8	2.05 (1.23-3.41)	2.00 (1.14-3.49)
		Neither condition	2.9	[Referent]	[Referent]
Czerwinski 2012^26^ (PIH)		Migraine + asthma	17.2	2.98 (1.73-5.15)	2.55 (1.40-4.63)
		Migraine only	10.5	1.68 (1.22-2.31)	1.21 (0.85-1.74)
		Asthma only	7.6	1.19 (0.78-1.82)	1.03 (0.66-1.62)
		Neither condition	6.5	[Referent]	[Referent]
Czerwinski 2012^26^ (PIH or preeclampsia)		Migraine + asthma	23.3	3.07 (1.92-4.89)	2.69 (1.59-4.56)
		Migraine only	13.5	1.58 (1.19-2.08)	1.15 (0.84-1.59)
		Asthma only	12.6	1.45 (1.04-2.02)	1.28 (0.88-1.84)
		Neither condition	9.0	[Referent]	[Referent]
Prophet 2018^27^ (any preeclampsia)		HIV + sickle cell disease	12.9	NR	3.62 (1.51-8.69)
		HIV only	3.5	NR	0.86 (0.78-0.96)
		Sickle cell disease only	5.3	NR	1.20 (1.10-1.30)
		Neither condition	3.5	[Referent]	[Referent]
Prophet 2018^27^ (mild preeclampsia)		HIV + sickle cell disease	6.3	NR	2.73 (0.97-7.66)
		HIV only	1.9	NR	0.76 (0.67-0.86)
		Sickle cell disease only	2.4	NR	0.93 (0.81-1.05)
		Neither condition	2.3	[Referent]	[Referent]
Prophet 2018^27^ (severe preeclampsia)		HIV + sickle cell disease	6.6	NR	4.28 (1.35-13.62)
		HIV only	1.7	NR	1.02 (0.90-1.16)
		Sickle cell disease only	2.7	NR	1.61 (1.44-1.79)
		Neither condition	1.2	[Referent]	[Referent]
Hospital transfer		Admon 2018 a^22^	≥ 2 chronic conditions	3.5	NR	NR
			1 chronic condition	2.0	NR	NR
			No chronic conditions	1.1	[Referent]	[Referent]
Perinatal emergency department visit		Varner 2020^28^ (1 visit)	7-32 ADGs	21.3	NR	1.97 (1.92-2.03)
			5-6 ADGs	22.8	NR	1.61 (1.59-1.64)
			3-4 ADGs	22.2	NR	1.33 (1.32-1.34)
			≤ 2 ADGs	19.7	[Referent]	[Referent]
		Varner 2020^28^ (2 visits)	7-32 ADGs	14.5	NR	2.78 (2.70-2.87)
	5-6 ADGs		13.7	NR	2.01 (1.98-2.04)
	3-4 ADGs		12.0	NR	1.49 (1.47-1.50)
	≤ 2 ADGs		9.5	[Referent]	[Referent]
	Varner 2020^28^ (3 visits)	7-32 ADGs	29.4	NR	7.59 (7.39-7.78)
		5-6 ADGs	18.0	NR	3.55 (3.49-3.61)
		3-4 ADGs	11.9	NR	1.99 (1.97-2.01)
		≤ 2 ADGs	7.1	[Referent]	[Referent]

Abbreviations: ADG = aggregated diagnosis group; CI = confidence interval; OR = odds ratio; NR = not reported; PIH = pregnancy-induced hypertension; RR = risk ratio.

### Quality of included studies

Study quality is described in Table 1. Studies were rated overall as strong (n=1),^32^ moderate (n=4),^28,30,31,33^ or weak (n=2)^27,29^ on the basis of study design, selection bias, confounding, detection bias, and attrition bias. Three of the studies were cross-sectional, leading to weak study design ratings.^27,28,30^ In terms of selection bias, one of the prospective cohort studies did not a report response rate and was rated as weak;^29^ the other had a response rate of 79% and was rated as moderate.^31^ The remaining studies were population-based. Two studies only controlled for 50% of the confounder categories identified *a priori* as being important and were rated as weak.^27,33^ The remainder controlled for most (n=4)^28,30,31,32^ or all (n=1)^29^ of the confounder categories. Regarding detection bias, all of the studies used established health administrative datasets and/or clinical data and were rated as moderate (n=5)^27,28,30,32^ or strong (n=2).^29,31^ Finally, neither of the prospective cohort studies reported follow-up rates,^29,31^ resulting in weak ratings for attrition bias for these studies.

### Synthesis of results

Studies generally found increasing risk of adverse maternal outcomes with an increasing number of chronic conditions. This was most clearly seen for severe maternal morbidity and mortality,^27,28,30^ hospital transfer,^27^ and emergency department visits.^33^ (Table 2). Admon et al.^27^ reported that rates of severe maternal morbidity and mortality were significantly higher in women with multimorbidity than those with one chronic condition or none, even after adjustment. The same associations were seen in a second study by Admon et al.,^28^ wherein results were stratified by race/ethnicity. In this study, the authors found the dose-response relation between the number of chronic conditions and risk of severe maternal morbidity was strongest among non-Hispanic Black women, followed by Hispanic, American Indian/Alaska Native, Asian/Pacific Islander, and White populations. Likewise, Brown et al.^30^ demonstrated a clear dose-response relation after adjustment between the number of chronic conditions (from 0 to ≥ 3 on the Elixhauser Comorbidity Index) and rates of severe maternal morbidity overall and without blood transfusion. Admon et al.^27^ also found a dose-response relation between the number of chronic conditions and rates of hospital transfer. Finally, Varner et al.^33^ showed a similar pattern for Johns Hopkins Adjusted Clinical Group System ADGs and risk of one, two, and three perinatal emergency department visits, after adjustment.

Findings were mostly similar for “any” pregnancy complication, and various definitions of preeclampsia. These latter outcomes were examined by studies of specific combinations of chronic conditions (Table 2). Prophet et al.^32^ found the odds of any pregnancy complication, preeclampsia, and severe preeclampsia were higher in women with sickle cell disease and HIV, compared to those with either or neither condition, after adjustment. Likewise, Czerwinski et al.^31^ showed individuals with migraine and asthma had the highest elevated odds of preeclampsia and pregnancy-induced hypertension, compared to individuals with either or neither condition, after adjustment. Only Bandoli et al.’s results did not support this sort of dose-response relationship, reporting that the risk of preeclampsia did not appear to be meaningfully greater in women with both autoimmune disease and depression, compared to those with autoimmune disease only or neither autoimmune disease nor depression.^29^

### Summary of evidence

The GRADE summary of evidence is shown in Table 3. The evidence for severe maternal morbidity and mortality was rated as having moderate certainty. While the evidence was downgraded due to methodological limitations, upgrades were also awarded because of large effects and the clear dose-response relationship between the number of chronic conditions and risks of the outcome. The evidence for perinatal emergency department visits was also upgraded to moderate certainty due to large effects and a clear dose-response relationship. The remaining outcomes were rated as having low or very low certainty due to downgrades resulting from methodological limitations and inconsistency.

**Table 3.** Summary of evidence using GRADE criteria.

Outcome	Effect	Number of participants (studies)	Certainty in the evidence*
Any pregnancy complication	0 studies reported significant effect	57,326,681 (1 study)	⊕⊕OO low certainty
Severe maternal morbidity or mortality	3 studies reported significant effects	6,015,264 (3 studies)	⊕⊕⊕O moderate certainty (downgraded due to methodological limitations; upgraded due to large effect and dose-response relation)^a^
Preeclampsia and related conditions (variously defined)	2 studies reported significant effects	57,333,522 (3 studies)	⊕OOO very low certainty (downgraded due to methodological limitations and inconsistency)^b^
Hospital transfers	1 study reported significant effect	1,508,413 (1 study)	⊕⊕OO low certainty
Perinatal emergency department visits	1 study reported significant effect	2,725,983 (1 study)	⊕⊕⊕O moderate certainty (downgraded due to methodological limitations; upgraded due to large effect and dose-response relation)^c^

* High certainty ⊕⊕⊕⊕, moderate certainty ⊕⊕⊕O, low certainty ⊕⊕OO, and very low certainty ⊕OOO.

^a^Risk of bias due to cross-sectional designs of 3 studies and lack of control for confounding for 1 study; however, studies showed strong effects and clear dose-response pattern.

^b^Serious risk of bias due to selection bias in 1 study and attrition bias in 2 studies, as well as inconsistency in findings across studies.

^c^Risk of bias due to unmeasured confounding; however, study showed strong effects and clear dose-response pattern.

## Discussion

### Main findings

In this systematic review, we identified seven studies examining the association between pre-pregnancy multimorbidity and adverse maternal outcomes in pregnancy and the postpartum period. Findings suggested that women with multimorbidity are at increased risk, compared to those with one or no chronic conditions, for severe maternal morbidity or mortality and emergency department visits in the perinatal period, with less certainty for other outcomes. Notably, only four of our studies defined multimorbidity using an adequate list of potential conditions; three studies examined specific comorbidities without consideration of other conditions. Several studies were excluded from our review because they examined one or more chronic conditions, or they included conditions that were not chronic (e.g., gestational diabetes). Given the high prevalence of multimorbidity in obstetric populations,^14-16^ as well as the preliminary findings of the studies included in this review, there is a need for more research examining the risks of adverse maternal outcomes in women with two or more conditions that pre-date the pregnancy, from among a comprehensive list of conditions.

### Comparison with existing literature

Our findings build on a growing body of literature in the general population showing that multimorbidity is associated with excess morbidity and mortality risk. Studies have shown that, across age groups, as the number of chronic conditions increases, the rate of acute health care use, length of hospital stay, and risk of mortality increases, and quality of life decreases.^5-7^ Outside of pregnancy, multimorbidity is also associated with high health care costs. One population-based study in Ontario, Canada, for example, found that multimorbidity accounts for 68% of overall health care costs.^7^ In individuals less than 65 years of age, having a second chronic condition (vs. 1) adds an extra $377 CAD in health care costs, and having a fifth (vs. 4) adds $2,073 CAD annually.^7^ A handful of studies have confirmed the rising prevalence of multimorbidity in obstetric populations. Using the National Inpatient Sample, Admon et al.^14^ found the prevalence of multimorbidity increased from 4.7 to 8.1 per 1,000 deliveries between 2005 and 2014. In a German study, Kersten et al.^15^ used self-reported and chart data on all chronic conditions from respondents to the cross-sectional Survey of Neonates in Pomerania to show that 5.3% of pregnant women had 2 chronic conditions, and 2.2% had 3 or more. Finally, in a study from the United Kingdom using three population-based primary care datasets, the prevalence of multimorbidity in the year before pregnancy ranged from 17.0% to 24.2%.^16^ However, despite clear importance in terms of frequency, the impact of multimorbidity on maternal outcomes has received minimal research attention.

### Reasons for findings

There are several mechanisms by which multimorbidity may increase the risk of adverse maternal outcomes relative to women with one or no chronic conditions. Potential biological mechanisms relate to the substantial physiological changes that accompany pregnancy, including cardiovascular, respiratory, and endocrine changes.^34^ Such changes have been shown to complicate the perinatal health of women with preexisting conditions,^35^ and may lead to even greater risk of complications in women with chronic conditions affecting more than one body system, particularly those with other risk factors, such as obesity, that may be more common in women with multiple chronic conditions. Further, women with multimorbidity may require multi-drug regimens that may interact in ways that are not well-understood in pregnant populations and could increase the risk of adverse side effects causing pregnancy complications.^36^ Finally, from a health systems perspective, models of health care delivery are typically oriented toward single, disease-specific processes and may be ill-equipped to address the complex interaction of multiple chronic conditions in pregnancy.^37^ In the perinatal period, this may lead to complications because of poor disease management. Further research is needed to explore these mechanisms in obstetric populations.

### Research implications

Our findings have implications for measurement of multimorbidity in obstetric research. While we identified several studies that examined the impact of co-occurring conditions on pregnancy outcomes, most failed to follow established definitions of multimorbidity.^18^ Thirty-seven studies used obstetric comorbidity indices^38^ that included both chronic conditions and conditions arising in pregnancy, such as gestational diabetes. While such studies provide data broadly relevant to the clinical care of women with complex pregnancies, they have limited utility for understanding how the growing burden of chronic disease impacts maternal health, which has implications for preconception counselling and disease management. Likewise, 25 studies were excluded because they examined “one or more” chronic conditions, making it impossible to discern the impact of *multiple* chronic conditions, whose combined effects could be very different than that of a single chronic condition. Because of the small number of studies using a strict definition of multimorbidity,^18^ we decided to also include studies examining specific comorbidities (e.g., asthma and migraine). While such studies are relevant to understanding specific co-occurring conditions and their clinical considerations (e.g., drug-drug interactions), they only hint at the issue of the dose-response relationship between the number of chronic conditions and risk of adverse maternal outcomes that is seen using comprehensive definitions of multimorbidity. In a systematic review of multimorbidity studies in the general population, Fortin et al.^18^ showed that use of different definitions of multimorbidity resulted in considerable heterogeneity in the reported prevalence of multimorbidity. They recommended the inclusion of at least 12 chronic conditions in definitions of multimorbidity, and the use of both “2 or more” and “3 or more” chronic conditions as the cut-off to identify the presence of multimorbidity. Such definitions could be applied in obstetric settings.

### Clinical implications

Internationally, preconception care guidelines emphasize the need to manage chronic conditions before pregnancy, with coordinated care across the perinatal period to avoid adverse outcomes.^39,40^ However, as is the case in the broader health care system,^41,42^ preconception and perinatal care are typically organized around the management of single chronic conditions.^41^ The increasing prevalence of risk factors for multimorbidity, including advanced maternal age and obesity,^44,45^ suggests that health care strategies to address the needs of women with multimorbidity will become increasingly important. Outside of pregnancy, collaborative, multidisciplinary approaches have been shown to be effective in the management of multimorbidity.^41^ These approaches include clinical guidelines and health care strategies developed to manage commonly co-occurring clusters of conditions, with particular attention to the biological mechanisms underlying the co-occurrence of these conditions as well as common issues such as drug-drug interactions.^42,43^ If more high-quality research confirms the findings of the current systematic review, these data would demonstrate the need for such care strategies for the management of multimorbidity in pregnancy and the postpartum period.

### Strengths and limitations

Strengths of our study include the comprehensive literature search using a validated search strategy for multimorbidity.^18^ However, we could have missed studies examining multiple chronic conditions if they did not use the terms included in our list of plain language keywords and controlled vocabulary. We initially planned to exclude studies that examined a specific comorbidity (e.g., asthma and migraine) without further evaluation of other conditions; however, an initial review of our search results showed few studies examining multimorbidity from among a long list of possible conditions. Within the scope of the search strategy, where specific combinations of comorbidities were identified, we thus also reported the outcomes for these studies. However, the search itself was not designed for examination of specific combinations of comorbidities. Our search was also restricted to papers written in English, which could have resulted in the exclusion of papers from developing countries where patterns and impacts of pre-pregnancy multimorbidity may differ. Given the heterogeneity in the studies identified, we were unable to conduct a meta-analysis, nor could we formally assess the risk of publication bias using a funnel plot. The studies included in our review were also limited by confounding and possible selection or attrition bias. Certainty of evidence was evaluated using the GRADE approach,^24^ modified for narrative syntheses.^25^ However, given the small number of studies available for any given outcome, no specific recommendations can be drawn.

## Conclusion

Our systematic review of seven studies suggests a dose-response relationship between the number of pre-pregnancy chronic conditions and risks of adverse maternal outcomes in pregnancy and postpartum. Given the increasing prevalence of chronic disease risk factors such as advanced maternal age and obesity, more research is urgently needed to understand the impact of multimorbidity on maternal health so that appropriate supports can be developed.

## Supplemental Material

Supplemental Material - Association between pre-pregnancy multimorbidity and adverse maternal outcomes: A systematic review Click here for additional data file. Supplemental Material for Association between pre-pregnancy multimorbidity and adverse maternal outcomes: A systematic review by Hilary K Brown, Anthony McKnight and Amira Aker in Journal of Multimorbidity and Comorbidity.
