# Supplementary material for: Association between pre-pregnancy multimorbidity and adverse maternal outcomes: A systematic review
Source: J Multimorb Comorb. 2022 Apr 30;12:26335565221096584. doi: 10.1177/26335565221096584 (PMC9106308; doi:10.1177/26335565221096584)
Supplement: Supplemental Material - Association between pre-pregnancy multimorbidity and adverse maternal outcomes: A systematic review [file sj-pdf-1-cob-10.1177_26335565221096584.pdf]

Table S1. Example of Medline search strategy

Concept	Keywords	MeSH terms
Multimorbidity[17]	Co-morbidity.tw,kf<br>Multimorbidity.tw,kf<br>Multi-morbidity.tw,kf<br>(Multiple adj2 (disease* or disorder* or condition*)).tw,kf<br>Comorbidity.tw,kf	Comorbidity/<br>Multiple chronic conditions/<br>Multimorbidity/
Maternal morbidity	((Maternal or perinatal or peripartum or prenatal or antenatal or pregnancy or postpartum or postnatal) adj2 complication*).tw,kf<br>((Maternal or perinatal or peripartum or prenatal or antenatal or pregnancy or postpartum or postnatal) adj2 morbidity).tw,kf	Maternal health/Pregnancy complications/<br>Pregnancy, high risk/<br>Pregnancy outcome/
Maternal mortality	((Maternal or perinatal or peripartum or prenatal or antenatal or pregnancy or postpartum or postnatal) adj2 mortality).tw,kf	Maternal death/<br>Maternal mortality/
Perinatal health care use	((Maternal or perinatal or peripartum or prenatal or antenatal or pregnancy or postpartum or postnatal) adj2 hospital*).tw,kf	Critical care/<br>Health care costs/<br>Hospitalization/<br>Intensive care units/<br>Length of stay/

Table S2. Study characteristics

Study	Study location, study period	Study design, data sources	Sample size, response, follow-up rates	Inclusion criteria	Multimorbidity definition	Outcome definition(s)	Confounders controlled for
Admon 2018 a[22]	United States, 2013-2014	Cross-sectional study, National Inpatient Sample	N=12,567 with ≥ 2 conditions<br>N=127,350 with 1 condition<br>N=1,368,496 with no conditions<br><br>Response rate: NA<br>Follow-up rate: NA<br>Missing data: < 3%	All hospital deliveries	2+ of chronic heart disease, chronic hypertension, chronic kidney disease, chronic liver disease, chronic respiratory disease, HIV, pre-existing diabetes, substance use disorders using ICD-9-CM codes from delivery hospitalizations	Severe maternal morbidity (composite) or mortality, hospital transfer, length of stay, using hospital discharge data	Controlled for age, rural residence, insurance payer, median household income, hospital region, obstetric outcomes (in sensitivity analyses)
Admon 2018 b[23]	United States, 2012-2015	Cross-sectional study, National Inpatient Sample	N=55,404 with ≥ 2 conditions<br>N=326,852 with 1 condition<br>N=2,141,636 with no conditions<br><br>Response rate: NA<br>Follow-up rate: NA<br>Missing data: < 6%	All hospital deliveries	2+ of chronic heart disease, chronic hypertension, chronic kidney disease, chronic respiratory disease, HIV, depression, pre-existing diabetes, pulmonary hypertension, substance use disorders, systemic lupus erythematosus using ICD-9-CM codes from delivery hospitalizations	Severe maternal morbidity (composite), using hospital discharge data	Stratified by ethnicity; controlled for age, rural residence, primary insurance payer, median household income, and hospital region
Bandoli 2017[24]	United States and Canada, 2009-2016	Prospective cohort study, MotherToBaby Study	N=179 with 1 of the autoimmune diseases and depression<br>N=1,228 with autoimmune disease only<br>N=153 with	English or Spanish-speaking, singleton livebirths	Comorbid autoimmune disease (rheumatoid arthritis, Crohn's disease, or psoriasis) and depression, using self-report verified by medical record review	Preeclampsia, using self-report verified by medical record review	Controlled for age, socioeconomic status, race/ ethnicity, medical comorbidities, smoking, BMI,

Study	Study location, study period	Study design, data sources	Sample size, response, follow-up rates	Inclusion criteria	Multimorbidity definition	Outcome definition(s)	Confounders controlled for
			depression only<br>N=1,550 with no conditions<br><br>Response rate: NR<br>Follow-up rate: NR<br>Missing data: NR				gestational age at enrollment
Brown 2020[25]	United States, 2016-2017	Cross-sectional study, National Inpatient Sample	N=60,718 with ≥ 3 comorbidities<br>N=145,131 with 2 comorbidities<br>N=296,185 with 1 comorbidity<br>N=1,480,925 with 0 comorbidities<br><br>Response rate: NA<br>Follow-up rate: NA<br>Missing data: <1%	All hospital deliveries with no missing data	Number of chronic comorbidities, defined using the 29 Elixhauser comorbidity measures identified by the Healthcare Cost and Utilization Project, excluding coagulation deficiency, complicated hypertension, congestive heart failure, peripheral perivascular disease, and pulmonary circulation disorders (which were also included in outcome), using hospital discharge data	Severe maternal morbidity (composite, overall and excluding those with blood transfusion as the only indicator), using hospital discharge data	Controlled for age, race/ ethnicity, primary expected payer, median household income, location of residence, delivery type
Czerwinski 2012[26]	United States, 1996-2008	Prospective cohort study, Omega Study	N=112 with asthma and migraine<br>N=933 with 1 condition<br>N=2,686 with neither condition<br><br>Response rate: 79%<br>Follow-up rate: NR<br>Missing data: < 4%	English-speaking, ≥ 18 years, prenatal care < 20 weeks, planning to deliver at term in eligible hospital	Comorbid asthma and migraine, using self-report verified by medical record review	Pregnancy-induced hypertension and preeclampsia, from medical records	Controlled for age, parity, race/ ethnicity, marital status, medical comorbidities, smoking, BMI, multifetal pregnancy

Study	Study location, study period	Study design, data sources	Sample size, response, follow-up rates	Inclusion criteria	Multimorbidity definition	Outcome definition(s)	Confounders controlled for
Prophet 2018[27]	United States, 2002-2014	Retrospective cohort study, Nationwide Inpatient Sample	N=222 with sickle cell disease and HIV<br>N=127,954 with 1 condition<br>N=57,198,505 with neither condition<br><br>Response rate: NA<br>Follow-up rate: NA<br>Missing data: < 1%	All hospital deliveries	Comorbid sickle cell disease and HIV, using ICD-9-CM codes from delivery hospitalizations	Severe preeclampsia, mild preeclampsia, pregnancy complications (composite), using hospital discharge data	Controlled for age, income, insurance status, race/ethnicity, geographical location
Varner 2020[28]	Canada, 2002-2017	Retrospective cohort study, Ontario health administrative data	N=43,952 with 7-32 ADGs<br>N=139,728 with 5-6 ADGs<br>N=471,959 with 3-4 ADGs<br>N=2,070,354 with ≤2 ADGs<br><br>Response rate: NA<br>Follow-up rate: NA<br>Missing data: <31%	All recognized pregnancies among Ontario residents	Number of comorbidities before the clinical start of pregnancy, expressed as the total number of Aggregated Diagnosis Groups (ADGs) obtained with the Johns Hopkins Adjusted Clinical Group System	Any emergency department visit during pregnancy or up to 42 days postpartum, using hospital discharge data	Controlled for age, parity, income quintile, region of residence, immigrant status, antenatal care provider

Abbreviations: ADG = aggregated diagnosis group; NA = not applicable; NR = not reported.
